# Bioequivalence of a Fixed-Dose Combination Tablet of the Complete Two-Drug Regimen of Dolutegravir and Rilpivirine for Treatment of HIV-1 Infection

**DOI:** 10.1128/AAC.00748-18

**Published:** 2018-08-27

**Authors:** Rashmi Mehta, Allen Wolstenholme, Kristin Di Lullo, Caifeng Fu, Shashidhar Joshi, Herta Crauwels, Naomi Givens, Simon Vanveggel, Brian Wynne, Kimberly Adkison

**Affiliations:** a GlaxoSmithKline, Research Triangle Park, North Carolina, USA; b GlaxoSmithKline, Collegeville, Pennsylvania, USA; c PAREXEL International, Durham, North Carolina, USA; d GlaxoSmithKline, QSI, Bengaluru, Karnataka, India; e Janssen Research and Development, Beerse, Belgium; f GlaxoSmithKline, Brentford, Middlesex, United Kingdom; g ViiV Healthcare, Collegeville, Pennsylvania, USA; h ViiV Healthcare, Research Triangle Park, North Carolina, USA

**Keywords:** two-drug regimen (2DR), dolutegravir, rilpivirine, fixed-dose combination (FDC), integrase strand transfer inhibitor (INSTI), nonnucleoside reverse transcriptase inhibitor (NNRTI), pharmacokinetics, bioequivalence

## Abstract

A complete 2-drug regimen of dolutegravir at 50 mg and rilpivirine at 25 mg was approved to treat HIV-1 infection in virologically suppressed patients after demonstrating acceptable efficacy and tolerability. This study investigated the bioequivalence and pharmacokinetics of the fixed-dose combination tablet compared with those of separate tablets. Secondary endpoints were the tolerability and safety of the fixed-dose combination tablet. In this open-label, randomized-sequence, 2-way crossover trial, single doses of the fixed-dose combination tablet (the test treatment) and the combination of separate tablets (the reference treatment) were administered to healthy adults after a moderate-fat meal, with a 21-day washout between treatments. Pharmacokinetic samples were collected through 12 days after dosing. The primary endpoints were the area under the plasma concentration-time curve (AUC) and the maximum concentration of drug in plasma (*C*_max_). The study employed a prespecified sample size reestimation based on a blind midpoint review of *C*_max_ variability to update the enrollment size to achieve statistical power. Of 118 participants enrolled, 113 received both treatments and underwent pharmacokinetic assessment. The 90% confidence intervals for the geometric least-squares mean ratios for the AUC from 0 h to infinity, the AUC from 0 h to the last quantifiable measurement, and *C*_max_ (test treatment versus reference treatment) were within the bioequivalence range of 0.80 to 1.25 for both drugs, indicating bioequivalence. In this study, a single dose of either treatment was well tolerated overall, with 4% (*n* = 5) and 3% (*n* = 3) of participants reporting adverse events considered related to the test and reference treatments, respectively. The dolutegravir-rilpivirine fixed-dose combination tablet is bioequivalent to a combination of separate tablets, and no new safety signals emerged. (This study has been registered at ClinicalTrials.gov under identifier NCT02741557.)

## Abstract (precis)

A complete 2-drug regimen of dolutegravir at 50 mg and rilpivirine at 25 mg was approved to treat HIV-1 infection in virologically suppressed patients after demonstrating acceptable efficacy and tolerability. This study investigated the bioequivalence and pharmacokinetics of the fixed-dose combination tablet compared with those of separate tablets.

## INTRODUCTION

Two ongoing phase III studies (SWORD-1 [ClinicalTrials.gov identifier NCT02429791] and SWORD-2 [ClinicalTrials.gov identifier NCT02422797]) provided evidence that a once-daily two-drug regimen (2DR) of the integrase strand transfer inhibitor (INSTI) dolutegravir (DTG) at 50 mg and the nonnucleoside reverse transcriptase inhibitor (NNRTI) rilpivirine (RPV) at 25 mg could maintain virologic suppression in patients with HIV-1 infection (1). In these studies, the drugs were administered as separate tablets. In a pooled analysis of the 2 study populations at week 48, 95% of participants had plasma HIV-1 RNA levels of <50 copies/ml, regardless of whether they received the 2DR of DTG and RPV or remained on their current antiretroviral therapy (ART) consisting of 2 nucleoside reverse transcriptase inhibitors (NRTIs) plus a protease inhibitor (PI), INSTI, or NNRTI. In addition to demonstrating noninferior virologic efficacy, the 2DR's safety and tolerability profiles were consistent with those found in previous studies that evaluated DTG (2–7) and RPV (8, 9). Formulation development of a fixed-dose combination (FDC) tablet of DTG at 50 mg plus RPV at 25 mg occurred in parallel with the SWORD studies, and the 2DR FDC tablet was approved for the treatment of HIV-1 infection in virologically suppressed patients in 2017 (10).

Prolonged use of ART can involve an increased onset of comorbidities, age-related vulnerability to ART-related toxicities, drug-drug interactions, and age-related obstacles to treatment adherence (11). In addition, the NRTIs that comprise 2 of the 3 components of every recommended treatment regimen (World Health Organization, http://apps.who.int/iris/bitstream/handle/10665/208825/9789241549684_eng.pdf; U.S. Department of Health and Human Services, https://aidsinfo.nih.gov/guidelines) have been associated with a range of adverse renal (12), cardiovascular (13), and bone health (14) effects. The 2DR of DTG and RPV is expected to provide patients with a simplified NRTI-sparing option for ART that is expected to support consistent medication adherence and reduce the lifetime cumulative drug exposure. An FDC tablet of these agents would be expected to simplify dosing further and improve full-regimen adherence by decreasing the chance for partial compliance (e.g., running out of 1 of the tablets in a multitablet regimen).

The present study was conducted to serve as a pharmacokinetic (PK) bridge between the approved FDC tablet and the separate tablets of DTG at 50 mg plus RPV at 25 mg, which were coadministered under fed conditions in the phase III trials (1). Rilpivirine is required to be taken with a meal to ensure optimal absorption, based on the approximately 40% decrease in absorption observed when this drug is administered after fasting versus the level of absorption after a normal-fat meal (15, 16). Therefore, the recommended intake for the 2DR is also with a meal (1). This is different from other DTG-based regimens (12), which can be administered regardless of food, despite the increase in DTG plasma exposure in the fed state (17).

The primary objective of this study was to evaluate the bioequivalence of the FDC and the 2 drugs as separate tablets by assessing plasma pharmacokinetic parameters under fed conditions. Secondary objectives of the study were to assess the pharmacokinetic parameters, safety, and tolerability of the FDC tablet. Moreover, this study employed a sample-size reestimation method (18) based on a blind (for treatment) midpoint review of pharmacokinetic variability estimates to update the enrollment size needed to achieve the targeted statistical power.

## RESULTS

### Participant characteristics.

One hundred eighteen participants were enrolled and randomly assigned to receive the reference or test treatment in period 1 followed by the alternate treatment in period 2. The mean age was 30.7 years, and most participants were male (69%) and white (69%) or black or African American (25%) (Table 1). The reference treatment was administered to 116 participants, and the test treatment was administered to 115. A total of 113 (96%) participants completed both treatment periods. Of the 118 participants in the safety population, 3 (3%) withdrew consent and 2 (2%) were withdrawn by physician decision. Twenty-nine (25%) participants received concomitant medications during the study, but none of the medications were expected to interfere with the study assessments. Forty-two protocol deviations occurred during the study, but none involved inclusion/exclusion criterion deviations or required exclusion from the pharmacokinetic analyses. Five of these events were considered important protocol deviations: use of excluded medications (*n* = 3), out-of-window PK sample collection (*n* = 1), and deviations in biological sample specimen procedures (*n* = 1).

**TABLE 1:** Participant demographics

Characteristic	Values (*n* = 118)
Mean (SD) age (yr)	30.70 (9.46)
No. (%) of participants by sex	
Female	36 (31)
Male	82 (69)
No. (%) of participants by ethnicity	
Hispanic or Latino	10 (8)
Not Hispanic or Latino	108 (92)
No. (%) of participants by race	
White/Caucasian	82 (69)
Black or African American	30 (25)
Multiple	4 (3)
American Indian or Alaska Native	1 (<1)
Native Hawaiian or other Pacific Islander	1 (<1)
Mean (SD) BMI^*a*^ (kg/m^2^)	26.21 (3.31)
Mean (SD) wt (kg)	78.84 (12.52)

a BMI, body mass index.

### Bioequivalence and pharmacokinetic parameters.

**FIG 1:**
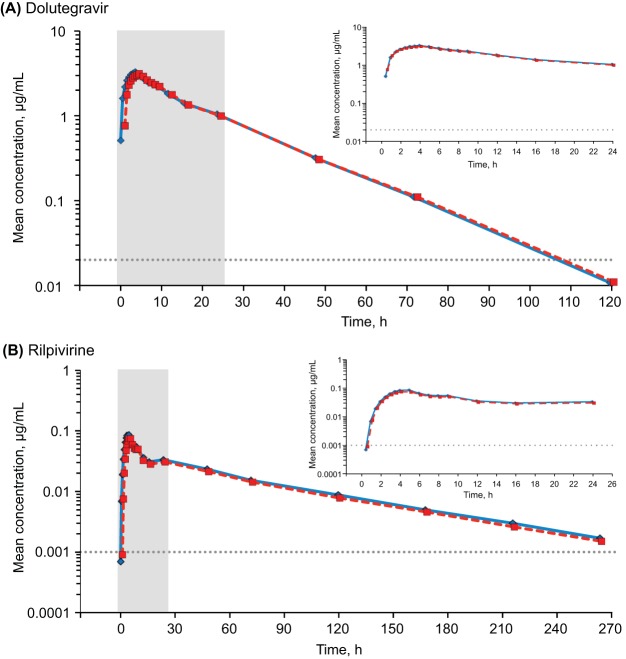
Mean plasma concentrations of DTG (A) and RPV (B) plotted on a semilogarithmic scale by time after dosing. The main graphs show the plasma concentrations through the full PK sampling time course. Insets show expanded views of the shaded areas, which represent the first 24 h of PK sampling. The first concentration in each plot corresponds to the PK sampling at 0.5 h after dosing. Dotted gray lines denote the lower limits of quantification, which were 0.02 μg/ml for DTG and 0.001 μg/ml for RPV.

The mean concentration-time curves associated with either DTG or RPV analytes were similar between the reference and test treatments (Fig. 1). The area under the concentration-time curve (AUC) from 0 h to infinity (AUC_0–∞_), the AUC from 0 h to the last quantifiable measurement (AUC_0–*t*_), the maximum concentration of drug in plasma (*C*_max_), and the plasma concentration at 24 h postdose (*C*_24_) for both the DTG and RPV analytes yielded adjusted geometric mean ratios that were close to 1, with 90% confidence intervals (CIs) being within the prespecified bioequivalence range of 0.80 to 1.25 (Table 2). Additional pharmacokinetic parameters are summarized with descriptive statistics in Table 3 and were consistent with the similar pharmacokinetic profiles between the DTG and RPV separate tablets and the DTG-RPV FDC tablets.

**TABLE 2:** Statistical analysis of log-transformed DTG and RPV PK parameters^*c*^

Analyte and PK parameter	Adjusted geometric mean (*n*) with the following treatment:		Test treatment/reference treatment ratio (90% CI)
	Test (DTG-RPV FDC tablet)	Reference (DTG and RPV separate tablets)	
DTG			
AUC_0–∞_ (μg · h/ml)	64.968 (113)	62.655 (113)	1.037 (1.010, 1.064)
AUC_0–_*_t_* (μg · h/ml)	63.583 (113)	61.265 (113)	1.038 (1.011, 1.066)
*C*_max_ (μg/ml)	3.646 (113)	3.474 (113)	1.050 (1.022, 1.078)
*C*_24_^*a*^	1.001 (112)	0.958 (112)	1.044 (1.012, 1.077)
RPV			
AUC_0–∞_ (μg · h/ml)^*b*^	3.248 (112)	2.933 (112)	1.108 (1.045, 1.174)
AUC_0–_*_t_* (μg · h/ml)	3.062 (113)	2.767 (113)	1.107 (1.042, 1.176)
*C*_max_ (μg/ml)	0.093 (113)	0.083 (113)	1.124 (1.047, 1.207)
*C*_24_	0.031 (113)	0.028 (113)	1.101 (1.034, 1.173)

a Paired data only; 1 participant was excluded because of a missing result in period 2.

b Paired data only; 1 participant was excluded because in period 1 the percentage of AUC_0–∞_ that was extrapolated was >20%, *R*^2^ was <0.85 in estimation of the terminal-phase rate constant, and the range of time over which the half-life (*t*_1/2_) was calculated was <2 × *t*_1/2_.

c AUC, area under the concentration-time curve; AUC_0–_*_t_*, AUC from 0 h to the last quantifiable measurement; AUC_0–∞_, AUC from 0 h to infinity; *C*_24_, plasma concentration at 24 h postdose; CI, confidence interval; *C*_max_, maximum concentration of drug in plasma; DTG, dolutegravir; FDC, fixed-dose combination; RPV, rilpivirine; *n*, number of participants.

**TABLE 3:** Summary of additional PK parameters based on actual sampling times^*a*^

PK parameter	DTG (*n* = 113)		RPV (*n* = 113)	
	Test (DTG-RPV FDC tablet)	Reference (DTG and RPV separate tablets)	Test (DTG-RPV FDC tablet)	Reference (DTG and RPV separate tablets)
Median (range) *T*_max_ (h)	3.02 (0.50, 6.00)	3.00 (0.50, 8.00)	4.00 (1.00, 9.00)	4.00 (1.50, 9.00)
Geometric mean (95% CI) AUC_0–24_ (μg · h/ml)	43.9 (42.3, 45.6)	42.4 (40.9, 44.1)	0.946 (0.885, 1.01)	0.860 (0.806, 0.919)
Median (range) *T*_lag_ (h)	0.00 (0.00, 1.03)	0.00 (0.00, 1.00)	0.50 (0.00, 2.50)	0.50 (0.00, 2.57)
Geometric mean (95% CI) CL/*F* (liters/h)	0.77 (0.74, 0.81)	0.80 (0.76, 0.84)	7.68 (7.12, 8.29)	8.53 (7.88, 9.22)
Geometric mean (95% CI) *t*_1/2_ (h)	14.5 (14.0, 15.1)	14.8 (14.2, 15.3)	51.7 (48.1, 55.7)	52.5 (48.8, 56.5)

a AUC, area under the concentration-time curve; AUC_0–24_, AUC from time zero to 24 h; CI, confidence interval; CL/*F*, apparent oral clearance; *C*_max_, maximum concentration of drug in plasma; DTG, dolutegravir; FDC, fixed-dose combination; RPV, rilpivirine; *t*_1/2_, half-life; *T*_lag_, absorption lag time; *T*_max_, time to *C*_max_.

### Safety and clinical evaluations.

The safety results were comparable, with adverse events (AEs) being reported in 17% and 18% of participants after the test and reference treatments, respectively (Table 4). Adverse events that occurred in 2 or more participants in any treatment group were headache, upper respiratory tract infection, contact dermatitis, and arthropod bite. Adverse events considered related to study medication were headache, diarrhea, catheter site swelling, and decreased appetite and were grade 1 (mild). One female participant was withdrawn from the study by physician decision after developing a grade 2 AE of bronchitis on day 2 following dosing with the DTG-RPV FDC tablet. The bronchitis resolved within 27 days of onset after palliative and antibiotic treatment, and the participant did not receive study medication during period 2. No deaths, serious AEs, grade 3 or 4 AEs, or other significant AEs were reported.

**TABLE 4:** Number of participants reporting adverse events^*d*^

AE	No. (%) of participants reporting AEs with the following treatment:	
	Test (DTG-RPV FDC tablet) (*n* = 115)	Reference (DTG and RPV separate tablets) (*n* = 116)
Any AE	20 (17)	21 (18)
AEs reported in ≥2 participants in either treatment group		
Headache	5 (4)	2 (2)
Upper respiratory infection	2 (2)	1 (<1)
Contact dermatitis	1 (<1)	2 (2)
Arthropod bite^*a*^	2 (2)	0
Any drug-related AE	5 (4)	3 (3)
Headache	4 (3)	2 (2)
Diarrhea	1 (<1)	0
Catheter-site swelling^*b*^	0	1 (<1)
Decreased appetite	1 (<1)	0
AEs leading to discontinuation of study drug	1 (<1)^*c*^	0

a A spider bite that became a localized methicillin-resistant Staphylococcus aureus infection.

b Related to study procedures and not a study drug.

c Grade 2 bronchitis on day 2 postdose; the bronchitis resolved 27 days after onset and was considered nonserious, moderate, and not related to the investigational product. The participant did not receive study drug in period 2 and was withdrawn from the study on day 36 by physician decision.

d AE, adverse event; DTG, dolutegravir, FDC, fixed-dose combination; RPV, rilpivirine.

### Clinical laboratory evaluations.

No laboratory abnormalities were reported as AEs. No participant was noted to have treatment-emergent toxicity grade increases from baseline in clinical chemistry abnormalities. One participant experienced elevated creatinine phosphokinase levels on day −1 of the second study period, but this event was attributed to the participant's vigorous exercise on the day before and was considered unrelated to study medication. No treatment-related or clinically significant hematology abnormalities were reported, and no participant had grade 2 or higher hematology abnormalities at baseline or after treatments. No clinically significant urinalysis or electrocardiogram (ECG) abnormalities were noted. No vital signs reported outside normal ranges were considered to be AEs by the investigators.

## DISCUSSION

The main objective of this study was to evaluate the bioequivalence between the DTG-RPV FDC tablet and the DTG and RPV tablets coadministered separately by assessing the plasma DTG and RPV AUC_0–∞_, AUC_0–*t*_, and *C*_max_ in the presence of a moderate-fat meal. The results of this study showed that for both DTG and RPV, the 90% CIs for the ratios of the adjusted geometric means for AUC_0–∞_, AUC_0–*t*_, and *C*_max_ were all fully contained within the bioequivalence limits (range, 0.80 to 1.25), indicating that the DTG-RPV FDC was bioequivalent to DTG and RPV as separate tablets. In addition, the median absorption lag times and times to *C*_max_ for both DTG and RPV were similar between the test and reference treatments.

Dosing with the DTG-RPV FDC tablet and DTG and RPV separate tablets did not lead to noted differences in reported AEs or laboratory changes, and both treatments were generally well tolerated. No grade 3 or 4 AEs, serious AEs, or deaths were reported during the study, and no treatment-related or clinically significant changes in clinical laboratory assessments were observed. One AE (bronchitis) led to discontinuation but was not considered by the investigators to be related to study medication. The safety results for both single-dose treatments were consistent with previous experience with DTG and RPV tablets (16, 19), with no new safety signals associated with the DTG-RPV FDC tablet being observed.

This study employed a recently reported sample size reestimation approach (18) to estimate the within-participant variability in the observed *C*_max_ parameters in a blind fashion while the study was ongoing. This interim analysis determined that the variability in the RPV *C*_max_ was higher than originally anticipated and that a greater number of participants (110 evaluable) would be needed to ensure that the prespecified statistical power of 90% was maintained.

### Conclusions.

Fixed-dose combination tablets containing complete ART regimens have become widely available and are considered an important option to support treatment simplification and patient convenience. Therefore, the availability of a complete, NRTI-sparing DTG-RPV FDC tablet that is bioequivalent to DTG and RPV separate tablets under fed conditions will provide a valuable new option in the treatment of HIV-1 infection. This study served as a pharmacokinetic bridge from the DTG-RPV FDC tablet to the ongoing phase III SWORD trials in which participants take DTG and RPV as separate tablets with a meal.

## MATERIALS AND METHODS

### Study design and participants.

**FIG 2:**
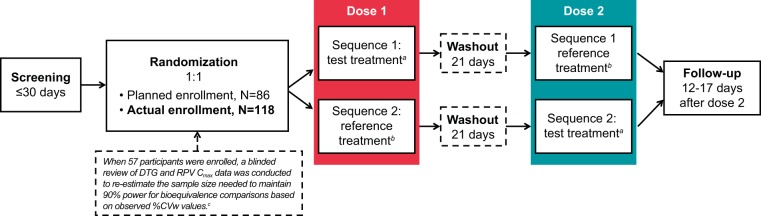
Two-way crossover study design. *C*_max_, maximum concentration of drug in plasma; CVw, within-participant coefficient of variation; DTG, dolutegravir; RPV, rilpivirine. ^*a*^, the test treatment was an FDC tablet containing DTG at 50 mg and RPV at 25 mg; ^*b*^, the reference treatment was separate tablets of DTG at 50 mg and RPV at 25 mg; ^*c*^, sample size reestimation determined that ≥110 evaluable participants would be needed to maintain a 90% power; 118 participants were enrolled to maintain 90% power and account for the possibility of participant discontinuations.

This phase I study (ViiV-clinicalstudyregister.com identifier 201676) utilized an open-label, randomized, 2-way crossover design at a single center (Quintiles, Overland Park, KS, USA) (Fig. 2). The total duration of the study was approximately 8 weeks from the time of screening to the last follow-up visit. A crossover design was selected for this study to allow within-participant comparisons to be performed, thereby reducing the number of participants required for the study. Rilpivirine has a long half-life (∼50 h) (15); therefore, a 21-day washout between doses and a long pharmacokinetic sampling period were implemented to ensure that predose concentrations were negligible and that pharmacokinetic parameters could be well estimated.

Eligible participants were healthy adults between 18 and 55 years of age, with body weights being ≥50 kg for men and ≥45 kg for women and the body mass index ranging from ≥18.5 to ≤31 kg/m^2^. Participants were excluded if they had evidence of alanine aminotransferase and bilirubin levels >1.5 times the upper limit of normal, a QT interval corrected by Fridericia's formula of >450 ms, a creatinine clearance of <90 ml/min, and/or a history of alcohol consumption amounting to >14 drinks per week for men or >7 drinks per week for women within 6 months of the study. The first participant was enrolled on 11 May 2016, and the last participants completed the study on 24 October 2016. The study design, participant enrollment criteria, procedures, and pharmacokinetic endpoints were consistent with the guidelines from the U.S. Food and Drug Administration (20) and the European Medicines Agency (21) on the conduct of bioequivalence studies. Informed written consent was obtained from all participants. The study was conducted in accordance with International Conference on Harmonisation of Technical Requirements for Registration of Pharmaceuticals for Human Use, good clinical practice, all applicable patient privacy requirements, and the ethical principles outlined in the Declaration of Helsinki 2013. The study protocol and informed consent document were reviewed and approved by a regional institutional review board (Midlands Independent Review Board, Overland Park, KS, USA). The study is registered with ClinicalTrials.gov under the identifier NCT02741557.

This study was designed to test the alternative hypothesis that the test treatment/reference treatment ratios of the pharmacokinetic parameter adjusted geometric means would fall within the bioequivalence criterion range of 0.80 to 1.25. For each pharmacokinetic parameter designated a primary endpoint (i.e., AUC_0–∞_, AUC_0–*t*_, *C*_max_), a two one-sided *t* test procedure (22) with α equal to 0.05 for each one-sided test was used to test the null hypothesis that the geometric mean ratio falls outside the bioequivalence range. Both the DTG and RPV analytes were required to demonstrate bioequivalence to conclude the bioequivalence of the FDC tablet and the 2 agents as separate tablets.

Initially, 86 healthy participants were planned to be enrolled and randomized to 1 of the 2 treatment sequences, which would provide a minimum of 82 evaluable participants and 4 additional participants to allow for dropouts. This target sample size of 82 evaluable participants was estimated to provide 90% power, based on the estimated within-participant coefficient of variation (CVw [in percent]) in the RPV maximum concentration of drug in plasma (*C*_max_; 28%), and an anticipated true treatment ratio of 1.10, based on preliminary drug interaction and relative bioavailability study data. A blind (for treatment) sample size reestimation was utilized after enrolling 57 participants to evaluate the appropriateness of the assumed PK parameter variability based on the observed percent CVw of the DTG and RPV *C*_max_ for these 57 participants (18). At the time of the reestimation, the observed percent CVw estimates for the DTG and RPV *C*_max_ were 14.8% and 32.9%, respectively. On the basis of the higher-than-anticipated RPV percent CVw, it was determined that ≥110 evaluable participants would be needed to maintain a 90% power for an anticipated true treatment ratio of 1.10. Thus, a total of 118 participants were enrolled to maintain a 90% power and account for the possibility of participant discontinuations. The sample size reestimation was performed by an independent statistician who was blind to the dosing sequence. No adjustments were made to the type I error rate because (i) no formal hypothesis testing was performed and (ii) the sample size reestimation was both variability based and done in a blind manner (i.e., the sequence of dosing for each subject was not known to the independent statistician).

### Treatments.

The reference treatment consisted of DTG 50-mg tablets (Tivicay; ViiV Healthcare, Research Triangle Park, NC, USA), which were identical in composition to the commercial form except for a different film coat color, and commercial RPV 25-mg tablets (Edurant; Janssen Therapeutics, Titusville, NJ, USA). The test treatment was the FDC tablet with DTG at 50 mg and RPV at 25 mg (Juluca; ViiV Healthcare, Research Triangle Park, NC, USA). A prior drug-drug interaction study of DTG at 50 mg once daily and RPV at 25 mg once daily with a moderate-fat meal showed no significant change in either the DTG or RPV AUC_0–*t*_ or *C*_max_ (23). Hence, the 2 drugs were combined in this study without dose adjustment.

Participants were randomly assigned 1:1 to 1 of 2 treatment sequences according to a randomization schedule generated before the start of the study. After randomization, participants took a single dose of either the reference or the test treatment during period 1 and then received the alternate treatment during period 2 after the 21-day washout period. Pharmacokinetic sampling was conducted at prespecified times before and after each treatment. Participants returned to the study center 12 to 17 days after period 2 for a follow-up visit.

All study treatments were administered after ∼10 h of fasting, followed by a standard moderate-fat breakfast scheduled at the same time of day in each period. Treatments were administered 30 (±5) min after the participants started eating breakfast. The meal consisted of approximately 625 cal, with 300 cal from carbohydrates, 200 cal from fat, and 125 cal from protein. Rilpivirine is recommended to be taken with a meal to ensure optimal absorption; therefore, treatments were administered in the fed rather than the fasting state, which is consistent with product label recommendations (10, 16, 19) and the dosing conditions employed in the SWORD-1 and SWORD-2 trials (1). Participants were instructed to abstain from consuming caffeine- or xanthine-containing products starting 24 h before dosing through the final pharmacokinetic sampling for each session and from taking prescription and nonprescription medications and dietary or herbal supplements, especially those known to alter the pharmacokinetics of either DTG or RPV (including histamine H_2_-receptor antagonists, proton pump inhibitors, antacids, vitamins, calcium, and iron) starting ∼7 days before dosing and for the duration of the study.

### Assessments.

Pharmacokinetic sampling was conducted for both drug analytes predose and at 0.5, 1, 1.5, 2, 2.5, 3, 3.5, 4, 5, 6, 7, 8, 9, 12, 16, 24, 48, 72, and 120 h after dosing. Additional samples, collected at 168, 216, and 264 h after dosing, were used to measure RPV only. Blood samples for measurement of DTG and RPV were taken via an indwelling cannula or direct venipuncture into dipotassium EDTA (K_2_-EDTA) and sodium heparin tubes, respectively. The tubes were agitated gently to mix the samples with the anticoagulants, stored at room temperature away from light for ≤45 min, and centrifuged for 10 min at 1,500 to 2,000 × *g* at 4°C. Plasma was transferred to fresh tubes within 30 min of centrifugation, frozen in an upright position at −20°C, and shipped from each site to the respective bioanalytical laboratory on dry ice.

Plasma samples were assayed for DTG and RPV using validated methods based on protein precipitation followed by ultraperformance liquid chromatography–triple-quadrupole mass spectrometry (23, 24). Assessment of the DTG analyte was conducted by PPD (Middleton, WI, USA), and assessment of the RPV analyte was conducted by PRA Health Sciences (Assen, the Netherlands). The methods could quantify DTG at concentrations of between 20 and 20,000 ng/ml in 25 μl of K_2_-EDTA-treated plasma and RPV at concentrations of between 1 and 2,000 ng/ml in 100 μl of heparin-treated plasma. Quality control (QC) samples containing DTG and RPV at prespecified concentrations were analyzed with each batch of samples against independently prepared calibration standards. For the analysis to be acceptable, no more than one-third of the QC results could deviate by >15% from the nominal concentration, and ≥50% of the results from each QC concentration had to be within 15% of the nominal concentration.

Safety assessments were conducted through the monitoring of AEs and concomitant medications throughout the study. A physical examination was conducted on the day before dosing during each study period. Clinical laboratory tests, ECGs, and pregnancy tests were performed on the day before dosing and during the follow-up period. Vital signs were monitored on the day before dosing, on the day of dosing (before and 4 h after dosing), at pharmacokinetic sampling times of between 24 and 120 h after dosing inclusive, and during the follow-up period.

### Pharmacokinetics and statistical comparisons.

Plasma DTG and RPV pharmacokinetic parameters were estimated by noncompartmental methods (based on actual sampling times) using WinNonlin (version 6.3) software (Certara USA, Inc., Princeton, NJ, USA) and summarized with descriptive statistics. The AUC_0–∞_, the AUC_0–*t*_, and *C*_max_ were designated the primary endpoints of the study. Following logarithmic transformation, AUC_0–∞_, AUC_0–*t*_, and *C*_max_ were separately analyzed for DTG and RPV using a mixed-effects model with fixed-effect terms for period and treatment and a random-effect term for participants. Logarithmic least-squares mean estimates and their 90% CIs were calculated for the differences between the test and reference treatments and then exponentially back-transformed to obtain adjusted (least-squares) geometric means for each treatment and point estimates and the associated 90% CIs for the test treatment/reference treatment ratio.

Safety data were summarized with descriptive statistics, and no formal statistical comparisons were conducted.
